# Genetic Influences and Targeted Treatments in Osteoporosis: A Systematic Review

**DOI:** 10.7759/cureus.87436

**Published:** 2025-07-07

**Authors:** Furkan Zurel, Hussain Bux, Aqeela Diveli, Zakaria Rashid

**Affiliations:** 1 Hospital Medicine, Salford Royal NHS Foundation Trust, Salford, GBR; 2 Hospital Medicine, Barking, Havering and Redbridge University Hospitals NHS Trust, London, GBR; 3 Hospital Medicine, University of Bolton, Bolton, GBR; 4 Hospital Medicine, Mid and South Essex NHS Foundation Trust, London, GBR

**Keywords:** anabolic drugs, anti-resorptive drugs, bisphosphonate use, genetic mutations, genome-wide association studies (gwas), osteogenesis, osteoporosis, polymorphisms, postmenopausal bone loss, postmenopausal osteoporosis

## Abstract

Osteoporosis is a chronic skeletal disorder marked by reduced bone mineral density (BMD) and increased fracture risk, posing a substantial global health burden. Traditionally considered multifactorial, growing evidence highlights a significant genetic contribution across both early-onset monogenic and adult-onset polygenic forms. Understanding the molecular and genetic architecture of osteoporosis is crucial for guiding targeted diagnostics and developing personalised therapeutic strategies.

This review aimed to: (1) identify and summarise genetic mutations and polymorphisms associated with osteoporosis, classifying them into monogenic and multifactorial causes; (2) distinguish between syndromic and non-syndromic forms of genetically influenced osteoporosis; (3) evaluate how specific genetic variations influence the risk, onset, and severity of osteoporosis, particularly in postmenopausal populations; (4) examine current anti-resorptive and anabolic treatments in the context of genetic backgrounds; and (5) identify gaps in knowledge to guide future research into genetics-based screening and individualised treatment.

A comprehensive literature search was conducted across PubMed, Embase, CINAHL, and the Cochrane Library for studies published between 2000 and 2025. Medical Subject Headings (MeSH) and free-text keywords were used to retrieve peer-reviewed articles, clinical trials, genetic association studies, and systematic reviews. Eligible studies explored genetic variants, bone signalling pathways (e.g., WNT/β-catenin, Notch), or pharmacological therapies in relation to BMD, fracture incidence, or osteogenesis. Data were extracted and thematically analysed under the following three core domains: genetic and molecular mechanisms, osteogenesis and bone remodelling, and treatment responses linked to genetic profiles.

The review identified a wide spectrum of genetic contributors to osteoporosis. Monogenic forms, often syndromic, were linked to mutations in genes such as *COL1A1*, *COL1A2*, and *WNT1*, whereas multifactorial osteoporosis, particularly postmenopausal, was associated with variants in *LRP5*, *SOST*, *VDR*, and other GWAS-identified loci. The interplay between these variants and osteogenic signalling cascades was found to influence bone homeostasis. Treatments were categorised as anti-resorptive (e.g., bisphosphonates, denosumab) or anabolic (e.g., parathyroid hormone analogues, romosozumab), with genetic factors influencing efficacy. The evidence suggests a future need for personalised therapeutic strategies based on genetic profiling.

There remains a need for further large-scale studies to validate genotype-phenotype correlations and treatment responses across diverse populations. Further exploration into pharmacogenomics, microRNA regulation, and gene-targeted interventions is required. Advancing osteoporosis care will depend on integrating genetic insights into clinical practice to enable earlier diagnosis, individualised treatment, and improved patient outcomes.

## Introduction and background

Osteoporosis is a globally prevalent disease and a significant public health burden [1]. It is characterised by low bone mineral density (BMD) and an increased risk of fractures, leading to substantial morbidity and healthcare costs [1,2]. Often asymptomatic until a fracture occurs, osteoporosis is frequently termed a “silent disease,” underscoring the importance of early detection and intervention [3].

While commonly linked to ageing and environmental factors, osteoporosis can also result from Mendelian monogenic disorders, with mutations in genes such as *COL1A1* and *COL1A2* impairing bone integrity [1]. The variability in disease onset and severity highlights the strong genetic influence on bone health. Failure to diagnose and treat osteoporosis early may result in chronic fractures, long-term disability, and a reduced quality of life [4]. With an ageing global population, the incidence of osteoporosis-related fractures is expected to rise, intensifying pressure on healthcare systems [5].

This systematic review aims to investigate the impact of current and emerging treatments on clinical outcomes in individuals with osteoporosis, including both early-onset monogenic and adult-onset multifactorial forms. It aims to assess how these interventions, compared to no treatment or standard care, influence BMD, fracture risk, and overall bone homeostasis. The review also explores the underlying genetic and cellular mechanisms driving osteoporosis to provide a comprehensive framework for evaluating therapeutic efficacy and identifying future directions for personalised disease management.

## Review

Methodology

**Figure 1:**
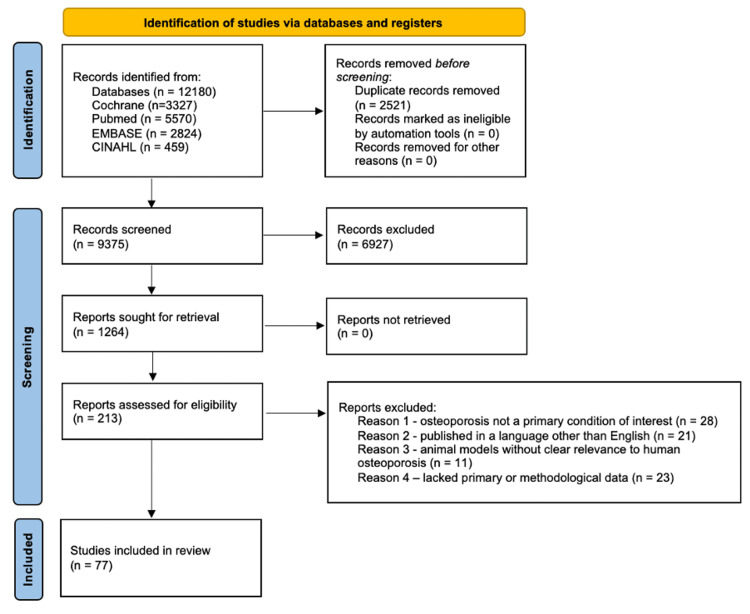
Preferred Reporting Items for Systematic Reviews and Meta-Analyses (PRISMA) flow diagram.

A systematic review was conducted to ensure a comprehensive evaluation of osteoporosis-related research. Relevant Medical Subject Headings (MeSH) terms were identified, and a structured search strategy was developed to capture high-quality studies on bone homeostasis, osteogenesis, and therapeutic interventions. This review was conducted in accordance with the Preferred Reporting Items for Systematic Reviews and Meta-Analyses (PRISMA) guidelines, as illustrated in Figure 1 [6].

Search Strategy

The search strategy was designed to reflect the multifaceted objectives of this review, including the identification and classification of genetic mutations and polymorphisms associated with osteoporosis, differentiation between syndromic and non-syndromic forms, analysis of genotype-phenotype correlations, and evaluation of genotype-informed therapeutic interventions. A targeted literature search was performed across four major databases, namely, the Cochrane Library, PubMed, Embase, and CINAHL, focusing on studies published between 1990 and 2025. The keywords and Boolean operators used in the search strategy included (1) genetic basis of osteoporosis (“osteoporosis” AND “genetic mutation” OR “genetic factors” OR “single nucleotide polymorphism” OR “monogenic” OR “multifactorial” OR “GWAS”); (2) syndromic vs. non-syndromic osteoporosis (“osteogenesis imperfecta” OR “monogenic osteoporosis” OR “multifactorial osteoporosis” AND “signalling pathways” OR “non-syndromic osteoporosis” OR “bone formation”); (3) bone metabolism and signalling pathways (“bone formation” OR “osteogenesis” AND “WNT signalling” OR “Notch pathway” OR “RUNX2” OR “RANKL”); (4) risk and severity factors in postmenopausal osteoporosis (“postmenopausal osteoporosis” AND “genetic predisposition” OR “oestrogen receptor polymorphism” OR “VDR gene”); and (5) genotype-guided treatment approaches (“osteoporosis” AND “treatments” AND “bisphosphonates” OR “denosumab” OR “romosozumab” OR “anabolic therapy” AND “genetic profile” OR “personalised medicine”).

Only peer-reviewed articles, clinical trials, genome-wide association studies (GWAS), and systematic reviews written in the English language were included. Studies were eligible if they addressed any of the review’s core objectives related to genetic underpinnings, classification, therapeutic implications, or treatment personalisation in osteoporosis. All selected articles were exported to EndNote for reference management. Duplicates were removed manually, and screening was performed according to eligibility criteria. Table 1 summarises the core concepts and corresponding search terms used to guide the literature search across selected databases.

**Table 1:** Search items.

Concept	Search terms
Osteoporosis	Bone density, bone loss, skeletal fragility, fractures
Genetic factors	*COL1A1*, *COL1A2*, *LRP5*, WNT signalling, GWAS findings
Bone Homeostasis	Osteoblasts, osteoclasts, bone remodelling, RANKL, OPG
Osteogenesis	Bone formation, mesenchymal stem cells, signalling pathways
Treatments	Bisphosphonates, denosumab, romosozumab, anabolic therapy, monoclonal antibodies

Eligibility Criteria

Table 2 outlines the inclusion and exclusion criteria used to guide the selection of studies.

**Table 2:** Inclusion and exclusion criteria.

Inclusion criteria	Exclusion criteria
Population: included individuals of any age diagnosed with osteoporosis or reduced bone mineral density, including both monogenic (e.g., osteogenesis imperfecta) and multifactorial forms (e.g., postmenopausal osteoporosis), as confirmed through clinical, radiological, or genetic criteria	Focused on diseases unrelated to osteoporosis or did not clearly define osteoporosis as a primary condition of interest
Genetic focus: investigated genetic mutations, single-nucleotide polymorphisms, or gene pathways (e.g., WNT/β-catenin, Notch, *RUNX2*, *COL1A1*, VDR) associated with the onset, severity, or progression of osteoporosis	Were published in a language other than English
Treatment focus: discussed current and future treatments for osteoporosis, including the impact of anti-resorptive (e.g., bisphosphonates, denosumab) or anabolic therapies (e.g., parathyroid hormone analogues, anti-sclerostin antibodies) on bone-related outcomes in relation to genetic profiles or stratified patient groups	Involved animal models without clear clinical or translational relevance to human osteoporosis
Outcomes: reported on at least one of the following: bone mineral density, fracture incidence, osteogenesis, or bone remodelling in relation to genetic or pharmacological factors	Lacked primary data or methodological transparency, such as letters, editorials, abstracts only, or studies with insufficient sample sizes or unclear outcomes
Study design: included both comparative and non-comparative studies: randomised controlled trials, cohort studies, case-control studies, genome-wide association studies, and relevant experimental or observational research	
Setting: conducted in clinical, laboratory, or academic research environments; studies from all geographical regions and income settings were considered	
Publication criteria: peer-reviewed, full-text articles published in the English language between 2000 and 2025.	

Study Selection

Two independent reviewers (F.Z. and H.B.) screened titles and abstracts, with full-text reviews conducted for eligible studies. Any disagreements regarding study inclusion were resolved through discussion between the two authors. Where a consensus was not reached, a third reviewer (A.D.) was consulted. One author (F.Z.) reviewed the reference lists of all included articles to identify additional relevant studies.

Data Extraction

The following data were extracted from the selected studies: study characteristics (author, year, study design, sample size); genetic and molecular findings related to osteoporosis; key signalling pathways involved in osteogenesis; and treatment strategies, including pharmacological and emerging genetic therapies.

Data Analysis

Extracted data were analysed using a thematic synthesis approach to systematically identify, organise, and interpret patterns within the literature. Studies were first grouped according to their primary focus and design (e.g., genetic association studies, signalling pathway analyses, pharmacological trials). Key themes were then derived iteratively through a detailed reading of included studies, with findings categorised under the following three key themes: (1) genetic and molecular basis of osteoporosis, including monogenic mutations, polymorphisms identified via GWAS, and gene function studies impacting bone metabolism; (2) osteogenesis and bone remodelling mechanisms, including cellular processes involving osteoblasts, osteoclasts, and osteocytes, as well as key signalling pathways such as WNT/β-catenin and Notch; and (3) genetic and pharmacological treatment strategies, including anti-resorptive and anabolic therapies, gene-targeted interventions, and emerging therapeutics.

Patterns of consistency and divergence across findings were highlighted to identify areas of strong evidence and gaps in the literature. This structured methodology facilitated a critical synthesis of current knowledge on the pathophysiology and management of osteoporosis.

Results

Study Characteristics

This review included a range of experimental and clinical studies investigating the genetic, molecular, and pharmacological aspects of osteoporosis. The methodologies encompassed GWAS, in vitro experiments, and clinical trials assessing osteoporosis treatments. These studies provided insights into genetic predisposition, signalling pathways involved in bone homeostasis, and pharmacological interventions aimed at improving BMD. A summary of key findings is presented in Table 3.

**Table 3:** Summary of key findings.

Study	Study type	Key findings
Experimental studies	Genetic manipulation	Loss of LRP5 function led to decreased bone mass in animal models [7,8]
Genome-wide association studies	Genetic analysis	Identified over 95 genetic loci associated with osteoporosis risk [9]
In vitro experiments	Laboratory research	Demonstrated the role of WNT signalling in osteoblast differentiation [10,11]
Clinical trials	Pharmacological studies	Denosumab was superior to bisphosphonates in BMD improvement [12]

Genetic and Molecular Basis of Osteoporosis

Numerous genetic factors were identified as contributing to osteoporosis susceptibility. *COL1A1* and *COL1A2* mutations were found to disrupt type I collagen synthesis, weakening bone structure [7]. Loss-of-function mutations in *LRP5* were associated with osteoporosis-pseudoglioma syndrome, highlighting the importance of the WNT signalling pathway [8]. Additionally, polymorphisms in genes such as *TNFRSF11B* (OPG) and *TNFSF11* (RANKL) were linked to variations in BMD and fracture risk [9]. GWAS studies identified over 95 genetic loci associated with osteoporosis risk, highlighting the complex genetic architecture of the disease.

Key Signalling Pathways in Osteogenesis

The review identified the following two primary signalling pathways critical to osteogenesis: (1) WNT/β-catenin signalling: this pathway regulates osteoblast differentiation and bone formation [9,10]. LRP5/6 activation enhances osteogenesis, whereas mutations in these genes impair bone accrual [8]. (2) Notch signalling: excessive Notch activity inhibits osteoblast maturation, contributing to bone loss. Studies indicate that a balance between Notch and WNT signalling is essential for maintaining bone homeostasis [11]. These findings emphasise the balance between signalling pathways in maintaining bone homeostasis.

Treatment Strategies for Osteoporosis

Osteoporosis treatments were categorised into anti-resorptive and anabolic therapies.

Anti-resorptive treatments: Bisphosphonates remain the first-line therapy, reducing osteoclast-mediated bone resorption. Denosumab, a monoclonal antibody targeting RANKL, was shown to increase BMD more effectively than bisphosphonates [12].

Anabolic therapies: Romosozumab (an anti-sclerostin monoclonal antibody) and parathyroid hormone analogues were found to promote bone formation, particularly in patients with postmenopausal osteoporosis (PMOP) [13,14].

Emerging therapies, including WNT pathway modulators, show potential for targeted interventions. However, concerns regarding long-term effects warrant further investigation.

Clinical Implications

The findings highlight the need for personalised treatment approaches based on genetic profiling. Advances in genetic modification techniques, such as CRISPR-based interventions, may offer potential therapeutic avenues for osteoporosis in the future.

Bone development

Osteogenesis

Bone development (osteogenesis) occurs through distinct biological processes. Importantly, the ability of bone to repair itself persists into adulthood, ensuring homeostasis [15]. However, this regenerative capacity declines with age, contributing to disorders such as osteoporosis [15]. Understanding the mechanisms underlying osteogenesis is crucial for developing effective treatments for bone diseases. Recent technological advancements have improved our comprehension of bone homeostasis and repair, influencing therapies for conditions such as osteoporosis [15]. However, despite these advances, challenges remain in translating this knowledge into clinical applications.

Osteogenesis is primarily driven by osteoblasts, which are responsible for producing essential components such as type I collagen and hydroxyapatite [16]. The transcription factor SOX9 initiates osteoprogenitor formation, while Runt-related transcription factor 2 (RUNX2) commits these cells to the preosteoblast stage [17]. Finally, WNT-β-catenin signalling triggers osterix (OSX) expression, finalising differentiation into osteoblasts. Notably, some osteoblasts become embedded within the bone matrix, transforming into osteocytes, which regulate bone homeostasis [18]. While these processes are well understood, external factors such as mechanical loading and hormonal fluctuations also influence osteoblast differentiation, raising potential areas for therapeutic intervention.

Osteocytes, the most abundant bone cells, serve as mechanosensors that mediate interactions between osteoblasts and osteoclasts [18-20]. During mechanical unloading, osteocytes express RANKL, activating osteoclasts and promoting bone resorption [21]. Conversely, during physical loading, osteocytes suppress DKK1 and sclerostin expression, enhancing WNT-β-catenin signalling to stimulate osteoblast activity. These opposing mechanisms maintain bone mass, but an imbalance, such as excessive osteoclast activation, leads to osteoporosis [20].

**Figure 2:**
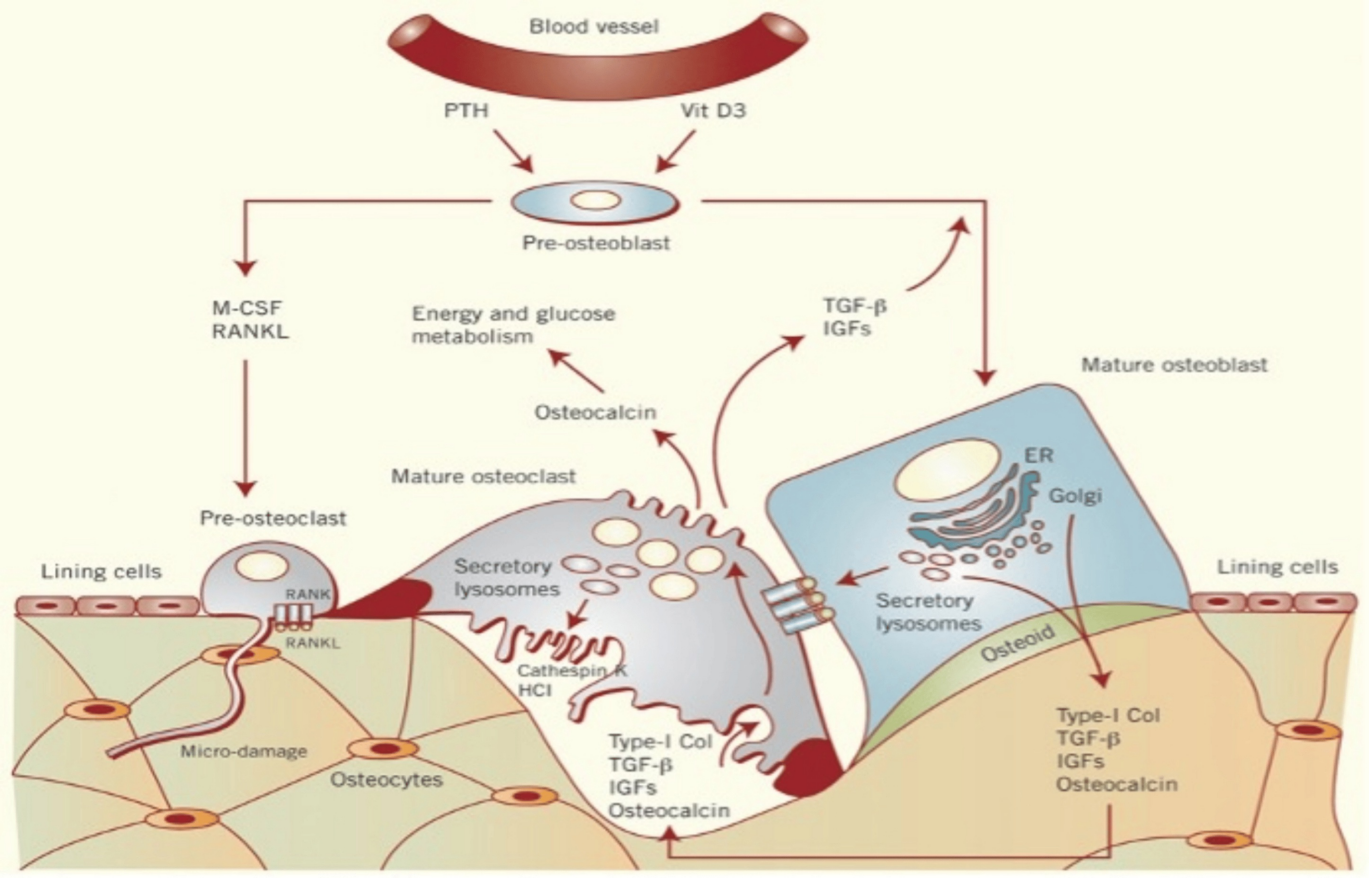
Macrophage colony-stimulating factor and RANKL interact to help differentiation into pre-osteoclasts which leads to mature osteoclast formation. Reproduced from Zhao [24] published under a Creative Commons License.

Osteoclasts, responsible for bone resorption, differentiate in response to macrophage colony-stimulating factor (M-CSF) and RANKL [22]. M-CSF drives osteoclast proliferation, while RANKL induces their maturation [22]. The balance between bone formation and resorption is crucial for homeostasis. Following resorption, transforming growth factor-β is released, stimulating osteoblast differentiation to replace old bone. However, excessive osteoclast activity disrupts this balance, leading to osteoporosis [23]. This is demonstrated in Figure 2 [24].

Osteogenesis Signalling Pathways

Signalling pathways play a critical role in bone homeostasis by regulating osteogenesis and resorption. Disruptions in these pathways contribute to skeletal disorders, making them key targets for therapeutic interventions.

**Figure 3:**
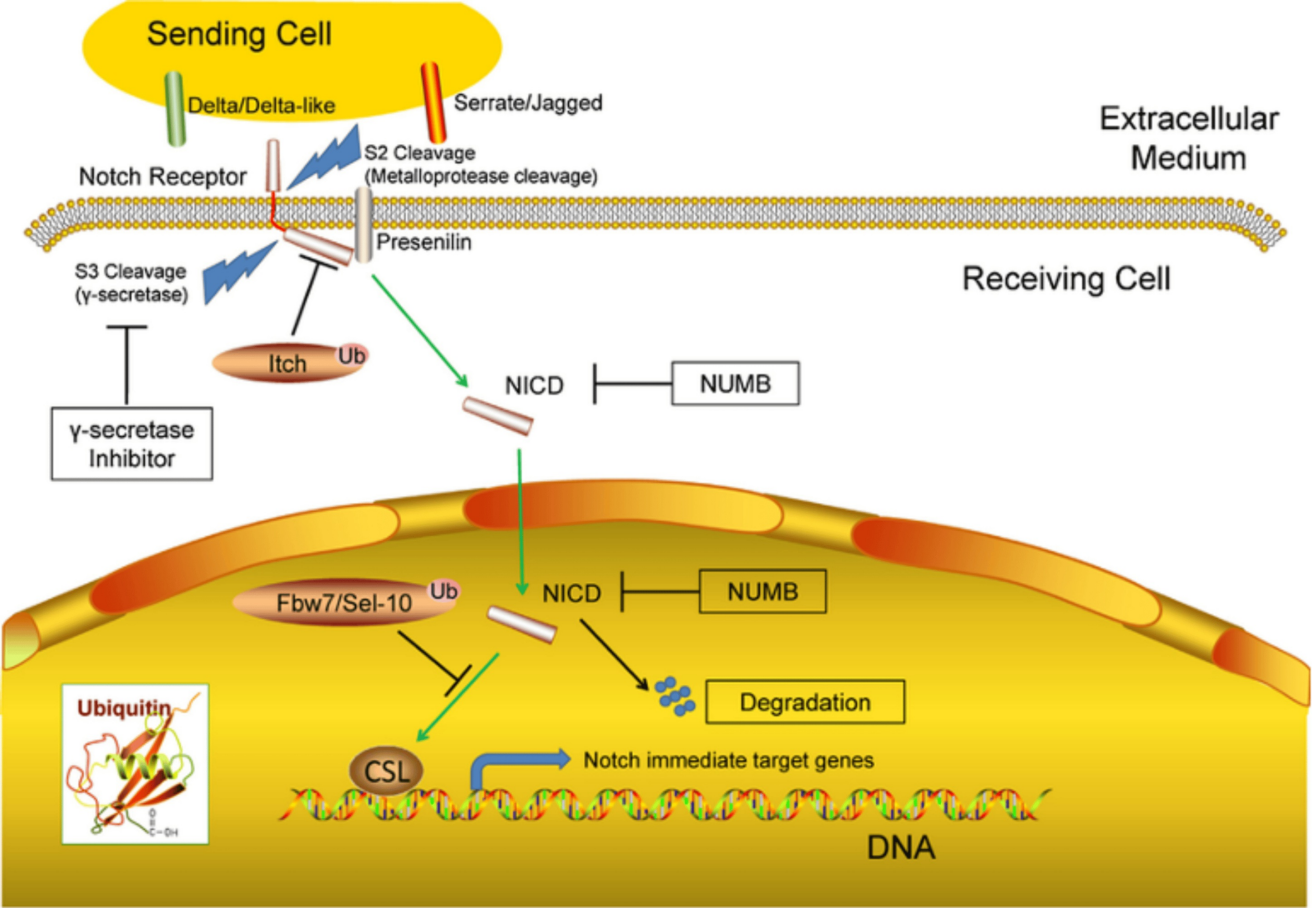
Summary of Notch intracellular domain transcription. Reproduced from Liu et al. [27] published under a Creative Commons License.

Notch signalling: Notch signalling involves direct contact between cells. Found on cells are Notch receptors (NOTCH1-4) which interact with other families, which leads to intracellular signalling [25]. This signalling, along with support from presenilin 1 (PS1) or PS2, causes proteolytic cleavage of the γ-secretase complex, which releases Notch intracellular domain (NICD) [26]. NICD is translocated to the nucleus, as shown in Figure 3 [27]. Here, it cooperates with hairless LAG-2 family transcription factor RBPJ, leading to the mastermind-like protein 1 (MAML1) activator complex, which further leads to the expression of Notch genes [25,26]. An increased expression of the *Notch1* gene in mice caused osteopenia because this prevented osteoblast cells from differentiating and specialising [18]. On the contrary, greater bone volume and osteoblast counts were recorded in mice with inactivation of *Notch1* and *Notch2* genes [28,29].

Canonical WNT signalling: In the canonical WNT signalling pathway, WNT ligands bind to a cell surface receptor and cause intracellular signalling [10]. Once activation occurs via WNT ligands binding to low-density lipoprotein receptor-related protein 5 (LRP5) or LRP6, β-catenin is stabilised [30-32]. This leads to β-catenin build-up in the cytoplasm, which enables its translocation to the nucleus where it interacts in transcription [15]. Loss-of-function mutations in LRP5 have been found to cause low bone mass and lead to osteoporosis-pseudoglioma syndrome, which presents with osteoporosis and eye defects [8]. Conversely, mutations that increase LRP5 function have been found to increase bone mass [33].

Following injury, WNT antagonists, for instance, DKK1, are upregulated to help bone repair by aiding osteoblast differentiation [15]. For instance, on day three of a fracture, upregulation of DKK1 was shown in mice [34]. On day 7, DKK1 was downregulated, emphasising how WNT signalling is temporary [34]. Additionally, the *SOST* gene codes for sclerostin and is upregulated in stem cells [35]. Sclerostin then binds to LRPs, silencing WNT activity. This means SOST acts as a negative regulator of osteogenesis and conflicts with the effects of LRP activation to prevent bone overgrowth [15]. Missense mutations in the *LRP5* gene produce a low binding affinity of LRP5 for sclerostin and DKK1, thus leading to high bone mass [36]. *LRP5* knockout in mice decreased bone mass, as seen in osteoporosis [33,37]. Likewise, haploinsufficiency of the *LRP6* gene in *LRP5*−/− mice also caused reduced bone mass [30]. The lower bone mass in *LRP5*-knockout mice is due to reduced osteogenesis; however, the lower bone mass in mice with haploinsufficiency of the *LRP6* gene in *LRP5*−/− is because of increased bone resorption [38].

The canonical WNT signalling pathway is a part of other signalling pathways too; the Notch pathway hinders the canonical WNT pathway and stops osteoblast differentiation [11]. As before, Notch pathway activation leads to greater osteoprogenitor proliferation but suppresses differentiation into mature osteoblasts. On the other hand, the canonical WNT pathway enables osteoblasts to develop into mature osteoblasts [15]. Ultimately, the balance between Notch signalling and canonical WNT signalling is vital in forming mature osteoblasts.

Osteoporosis

Bone homeostasis is maintained by a dynamic balance between osteoblast-mediated bone formation and osteoclast-driven bone resorption. However, ageing disrupts this equilibrium, leading to increased osteoclast activity and progressive bone loss [15]. This imbalance is particularly pronounced in postmenopausal women due to declining oestrogen levels, which further accelerate bone resorption [39]. Osteoporosis is a systemic skeletal disorder characterised by bone fragility and fractures, with prevalence increasing with age and disproportionately affecting women [40]. Approximately 200 million people suffer from osteoporosis worldwide [41,42].

The development of osteoporosis is influenced by both genetic and environmental factors. Large-scale GWAS have identified genetic variants linked to BMD and fracture risk, providing insights into the molecular mechanisms of osteoporosis. Interestingly, several of the pathways identified through GWAS overlap with those targeted by current osteoporosis treatments, suggesting a strong genetic basis for therapeutic response [43]. The following section explores the underlying causes of bone fragility in osteoporosis.

Causes of osteoporosis

Osteoporosis can be categorised into two primary types, namely, monogenic and multifactorial. Monogenic osteoporosis is typically childhood-onset and often presents with bone fragility alongside other abnormalities. In contrast, multifactorial osteoporosis is generally characterised by bone fragility alone and manifests later in life. The next part of this review will analyse the different causes of each.

Monogenic Osteoporosis

Monogenic osteoporosis can present as syndromic, where bone fragility is accompanied by other conditions, or non-syndromic, where bone fragility is the sole feature. It is primarily caused by single-gene mutations, as outlined in Table 4, with the majority being recessive (fourteen recessive, six dominant) [44]. These mutations often affect osteoblast or osteoclast function, collagen formation, bone development, or calcium release. Syndromic forms, such as gerodermia osteodysplastica (GO), caused by *GORAB* mutations, include additional symptoms such as skin wrinkling and premature ageing [44,45]. Table 4 also describes how mutations in collagen genes (*COL1A1* and *COL1A2*) cause osteogenesis imperfecta (OI), a disorder characterised by bone fragility due to defective collagen formation [7,46-87].

**Table 4:** Different genes that underlie the causes of different conditions that include bone fragility and reduced BMD. AR: autosomal recessive; AD: autosomal dominant; BMD: bone mineral density; XLD: X-linked dominant The table has been created by the authors.

Gene	Function	Chromosome location	Inheritance mode	Condition	Phenotypes	Citation
*GORAB*	Glycosylation of proteins. Formation of stable domains at the trans-Golgi. Aids in the transport of vesicles in osteoclasts	1q24.2	AR	GO	Bone fragility. Wrinkling of the skin. Greater ears. Prematurely aged	[44,48-51]
*CALCR*	Regulates osteoclast-mediated bone resorption. Sustains calcium homeostasis	7q21.3	AD	Osteoporosis	Reduced BMD	[52,53]
*LRP5*	Expressed in osteoblasts. Leads to the canonical WNT signalling pathway. Responsible for the inhibitory effects of DKK1 protein	11q13.2	AR	OPPG	Reduced BMD. Visual impairments	[52,54-56]
*RIL*	Osteoblast development and function. Bone formation	5q31.1	AD	Osteoporosis	Reduced BMD	[52,57,58]
*WNT1*	Involved in osteoblast function, bone development, and bone homeostasis	12q13.12	AR	Osteogenesis imperfecta. Early-onset osteoporosis	Reduced BMD. Bone fragility. Blue sclera. Hearing impairments	[46,59,60]
*SP7*	Essential for osteoblast differentiation and bone formation	12q13.13	AR	Osteogenesis imperfecta	Reduced BMD. Bone fragility. Blue sclera. Hearing impairments	[46,61,62]
*CRTAP*	Responsible for osteoblast invasion and organisation of collagen fibrils	3p22.3	AR	Osteogenesis imperfecta	Reduced BMD. Bone fragility. Blue sclera. Hearing impairments	[46,63,64]
*COL1A1*	Encodes alpha-1 chain for type 1 collagen in bone	17q21.33	AD	Osteogenesis imperfecta	Reduced BMD. Bone fragility. Blue sclera. Hearing impairments	[46,52,65]
*COL1A2*	Encodes alpha-2 chain for type 1 collagen in bone	7q21.3	AD	Osteogenesis imperfecta	Reduced BMD. Bone fragility. Blue sclera. Hearing impairments	[46,52,66]
*PPIB*	Involved in alpha-1 chains for type 1 collagen. Helps with protein folding	15q22.31	AR	Osteogenesis imperfecta	Reduced BMD. Bone fragility. Blue sclera. Hearing impairments	[46,67,68]
*P3H1*	Essential for collagen synthesis	1p34.2	AR	Osteogenesis imperfecta	Reduced BMD. Bone fragility. Blue sclera. Hearing impairment. Short stature. Recurrent fractures	[46,69,70]
*FKBP10*	Responsible for type 1 procollagen folding	17q21.2	AR	Osteogenesis imperfecta. Type 1 Bruck syndrome	Reduced BMD. Bone fragility. Blue sclera. Hearing impairment. Short stature. Scoliosis	[46,71,72]
*SERPINH1*	Essential for collagen synthesis	11q13.5	AR	Osteogenesis imperfecta	Reduced BMD. Bone fragility. Blue sclera. Hearing impairments	[46,73,74]
*SERPINF1*	Codes for proteins that bind to type 1 collagen. Regulates type 1 collagen function	17p13.3	AR	Osteogenesis imperfecta	Reduced BMD. Bone fragility. Blue sclera. Hearing impairments	[46,75,76]
*CREB3L1*	Essential for bone formation. Involved in COL1A1 and COL1A2 transcription and bone matrix protein secretion	11p11.2	AR	Osteogenesis imperfecta	Reduced BMD. Bone fragility. Blue sclera. Hearing impairments	[46,77,78]
*PLS3*	Involved in regulating bone development	Xq23	XLD	Osteoporosis	Increased fracture risk. Bone dysplasia	[79,80]
*IFITM5*	Involved in coding for proteins for bone mineralisation	11p15.5	AD	Osteogenesis imperfecta	Reduced BMD. Bone fragility. Blue sclera. Hearing impairments	[46,81,82]
*SPARC*	Involved in extracellular matrix synthesis	5q33.1	AR	Osteogenesis imperfecta	Reduced BMD. Bone fragility. Blue sclera. Hearing impairments	[46,83]
*TMEM38B*	Acts as a channel to synchronise the release of calcium from intracellular stores	9q31.2	AR	Osteogenesis imperfecta	Reduced BMD. Bone fragility. Blue sclera. Hearing impairments	[46,84,85]
*BMP1*	Essential for cartilage synthesis	8p21.3	AR	Osteogenesis imperfecta	Reduced BMD. Bone fragility. Blue sclera. Hearing impairments	[46,86,87]

Furthermore, the severity of monogenic osteoporosis can vary. For example, mutations in *LRP5* lead to either a dominant form of osteoporosis with reduced BMD or a recessive form, osteoporosis-pseudoglioma syndrome, which is more severe and associated with additional phenotypes [47]. This variability can be observed with other genes, such as *GORAB*, where a single mutation may lead to reduced BMD, while two mutations cause the more severe GO.

Multifactorial Osteoporosis

Multifactorial osteoporosis has been identified via GWAS, which uncover loci associated with increased susceptibility to osteoporosis. Unlike monogenic forms, where a single gene mutation leads to the condition, multifactorial osteoporosis results from the combined influence of multiple genetic factors. Ultimately, GWAS are used to identify increased susceptibility and to predict onset. Multifactorial osteoporosis tends to be later-onset and non-syndromic, thus involving bone fragility alone. The role of environmental factors, such as diet, is excluded from this section, although they are known to contribute to disease development. Postmenopausal women have been included. This is because not all are diagnosed with osteoporosis post-menopause, suggesting genetics has a key influence. However, hormonal and nutritional factors regarding this have been excluded. Many factors contribute to the cause of multifactorial osteoporosis, and each factor has a small contribution to the phenotype; having a combination of certain loci can predispose individuals to multifactorial osteoporosis.

Genetic variants in osteoporosis: Over 95 major genes have been discovered using GWAS; however, only 41 of the genes (shown in Table 5) have evidence supporting their involvement in bone physiology [9,88-91]. The remaining have been identified as contributing to osteoporosis via GWAS; however, further research is needed to understand their significance.

**Table 5:** Genes identified in GWAS that are known to influence BMD, along with their corresponding single-nucleotide polymorphisms. *: associated with monogenic disorders; ^†^: discussed in the text. The table has been adapted from Rocha-Braz et al. [9]. Source: GeneALaCart [91]. BMD: bone mineral density; GWAS: genome-wide association studies; TGF-β: transforming growth factor-beta

Gene	Corresponding SNP	Function	Citation
ABL1	rs7851693	Bone remodelling	[9,91]
AXIN1	rs9921222	β-catenin destruction and WNT signalling modulation	[9,91]
CLCN7	rs163879	Mediates chloride exchange for protons	[9,91]
CREB3L1*	rs7932354	Bone formation	[9,91]
CTNNB1	rs430727	Component of the canonical WNT signalling pathway	[9,91]
DKK1	rs1373004	Antagonises canonical WNT signalling by inhibiting LRP5/6 interaction with WNT	[9,91]
DMP1	rs6532023	Osteoblast differentiation	[9,91]
EN1^†^	rs11692564	Osteoblast function	[9,91]
ESR1^†^	rs4869742	Bone growth	[9,91]
FKBP11	rs12821008	Protein folding	[9,91]
FOXC2	rs10048146	Transcriptional activator	[9,91]
GALNT3	rs6710518	Glycosylates fibronectin	[9,91]
GPR68	rs1286083	Regulates cell-mediated responses to acidosis in bone	[9,91]
GREM2	rs9287237	Modulates signalling by BMP family members	[9,91]
IBSP	rs6532023	Forms part of the bone matrix	[9,91]
JAG1	rs1878526	Involved in Notch signalling (acts as a ligand)	[9,91]
LRP4	rs7932354	Facilitator of SOST-mediated inhibition of WNT signalling	[9,91]
LRP5*^†^	rs3736228	Osteoblast proliferation and differentiation	[9,91]
MECOM	rs784288	Involved in TGF-β signalling	[9,91]
MEPE	rs6532023	Aids bone mineralisation	[9,91]
NBR1	rs4792909	Receptor for degradation of ubiquitinated targets	[9,91]
PKDCC	rs7584262	Helps bone growth	[9,91]
PTHLH	rs7953528	Aids bone homeostasis	[9,91]
RSPO3	rs13204965	Activates the canonical WNT signalling pathway	[9,91]
RUNX2	rs11755164	Involved in osteoblast differentiation	[9,91]
SALL1	rs1566045	Organogenesis	[9,91]
SHFM1	rs4727338	degradation of ubiquitinated proteins	[9,91]
SMOC1	rs227425	Regulates osteoblast differentiation.	[9,91]
SOST	rs4792909	Negative regulator of bone growth. Inhibits WNT signalling and osteogenesis	[9,91]
SOX4	rs9466056	Transcription factor	[9,91]
SOX6	rs7108738	Osteogenesis	[9,91]
SOX9	rs7217932	Osteogenesis	[9,91]
SP7	rs2016266	Osteoblast differentiation	[9,91]
SPP1	rs6532023	Important in skeletal tissue	[9,91]
SUCO	rs479336	Regulates type I collagen synthesis in osteoblasts	[9,91]
TNFRSF11A (RANK)	rs884205	Osteoclast production	[9,91]
TNFRSF11B (OPG)^†^	rs2062377	Inhibits osteoclast activation	[9,91]
TNFSF11 (RANKL)^†^	rs9533090	Osteoclast differentiation	[9,91]
WLS	rs1430742	Regulates Wnt protein	[9,91]
WNT16	rs3801387	Ligand for members of the frizzled family	[9,91]
WNT5B	rs2887571	Ligand for members of the frizzled family	[9,91]

In one GWAS, key genes involved in osteoporosis were *TNFRSF11B* (*OPG*), *TNFSF11* (*RANKL*), *LRP5*, and *ESR1* (Table 5) [9]. As explained before, *RANKL* helps osteoclasts differentiate and become mature, and *LRP5* is important in initiating WNT signalling pathways for osteogenesis. *ESR1* is important in osteoporosis because of its role in bone growth and maintenance [88]. Scientists discovered the significance of EN1 in BMD in another GWAS [9]. EN1 is crucial for osteoblast function and indirectly affects osteoclast recruitment and bone resorption [89,90]. This GWAS focused on discovering low-frequency loci, such as EN1, and suggested that these can have greater influences on BMD and fracture risk [9,91].

Postmenopausal osteoporosis: Micro-RNA (miRNA) regulates several biological processes, including osteogenesis and bone resorption [92]. miRNA 133a leads to a downregulation of *RUNX2* expression, which is essential for regulating differentiation into osteoblasts [92]. This is found in PMOP due to a lack of oestrogen; therefore, miRNA 133a is most likely upregulated in PMOP [92]. Furthermore, miRNA 27 is downregulated in PMOP and miRNA 27 works by helping osteoblasts differentiate, which explains its contribution to PMOP [92]. As a result, screening for PMOP could involve analysing plasma miRNA levels to identify early diagnoses for a better prognosis [93].

In terms of PMOP susceptibility, scientists have researched the roles of vitamin D receptor (VDR) gene polymorphisms in PMOP and their effects on BMD.

The VDR ApaI polymorphism is important in PMOP and BMD because it alters vitamin D function and has a significant association with PMOP risk [94,95]. This polymorphism protects postmenopausal women from developing osteoporosis [94]. The aa genotype of VDR ApaI was greatly implicated in increased BMD [96,97]. As the VDR ApaI polymorphism is located in the non-coding region of VDR, further research is needed to identify how it affects BMD. Moreover, having different genotypes of this polymorphism could affect the BMD of individuals differently [94]. For example, PMOP women with the Aa genotype were not significantly different from PMOP women with the AA genotype [94].

The VDR BsmI polymorphism is important in maintaining VDR mRNA stability [94]. VDR BsmI is known to have strong associations with developing PMOP in Asian and overall populations [94]. No significant differences were reported between Bb genotypes and bb genotypes and between BB and bb genotypes [94]. Women with PMOP with the Bb or BB genotype were not at a considerably higher risk of low BMD than those with the bb genotype [98,99]. This could be because VDR BsmI does not affect the VDR amino acid sequence [94]. As there has been no discovery into the associations of the VDR BsmI polymorphism in Caucasian populations, ethnicity could be a major factor in PMOP predisposition; therefore, further studies are needed [94]. When compared with VDR ApaI, VDR BsmI was linked with a higher risk of PMOP and does not significantly affect BMD, whereas VDR ApaI is linked with a decreased risk of PMOP (due to its protective effects) and higher levels of BMD [94]. Although both polymorphisms impact VDR mRNA stability, their different effects could be due to the different locations of the genes; however, further studies are required to support this.

The VDR Cdx2 polymorphism is thought to be involved in calcium absorption and VDR activation to vitamin D [94]. The VDR Cdx2 polymorphism plays a protective role against PMOP and thus no link to PMOP in Caucasian populations, Asian populations, or overall populations was found [94]. However, women with PMOP with VDR Cdx2 polymorphism and the GA genotype were observed to have reduced BMD; having the GA genotype of VDR Cdx2 also gave greater chances of developing low BMD than the GG genotype [94,97]. With the AA genotype of VDR Cdx2, no significant difference in BMD was reported when compared with PMOP women with the GG genotype [94]. However, the sample sizes of these studies emphasise that studies with greater sample sizes are essential to help support these associations.

The VDR FokI polymorphism is important in message stability and post-transcriptional mechanisms [100]. This polymorphism is appreciably associated with a greater risk of developing PMOP in overall and Asian populations, but not in Caucasian populations [94]. In Caucasian populations, women with PMOP with the Ff genotype of VDR FokI had a considerably lower BMD than women with the FF genotype [94]. Moreover, the ff genotype was not shown to have strong associations with BMD [94]. This means that the Ff genotype contributes to decreased BMD in Caucasians. However, further research is essential to observe its role in other populations.

Treatments for osteoporosis

When treating osteoporosis, the causes must be understood because individualised treatment plans are crucial. The patient’s genetic background can determine which treatments are most effective for them. For instance, patients with GO, which involves defects in the *GORAB* gene affecting Golgi trafficking, can benefit from bisphosphonates.

Treatment for osteoporosis is categorised into two groups: anti-resorptive drugs, which help delay bone resorption, and anabolic drugs, which help with osteogenesis [13,101].

Anti-resorptive drugs, with bisphosphonates being the largest class, are commonly used to treat a range of conditions, including PMOP and GO [13]. They work by inhibiting osteoclast-mediated bone resorption by transforming to cytotoxic ATP analogues intracellularly [102,103]. Bisphosphonates bind to hydroxyapatite, a mineral component of bone, particularly in areas with higher bone turnover, such as trabecular bone [104,105]. Because of its greater surface area, trabecular bone can bind more bisphosphonate than cortical bone [106]. Some bisphosphonates are more efficient at binding to bone, while others penetrate bone tissue better [107]. Once bound to bone, bisphosphonates can be released during bone resorption, where they affect osteoclast function [104,105,108]. Bisphosphonates that contain nitrogen inhibit the enzyme farnesyl pyrophosphate synthase, which reduces osteoclast activity, ultimately inhibiting bone resorption [94]. Once bone resorption occurs, bisphosphonates are released into circulation. Bisphosphonates may then bind back onto bone depending on their properties [94].

Furthermore, bisphosphonates inhibit osteoclast formation by enhancing the release of inhibitory factors by decreasing RANKL expression [109]. Therefore, bisphosphonates interfere with RANKL signalling and, in doing so, interfere with the differentiation of osteoclasts, giving an anti-resorptive effect [109].

New bisphosphonates are being discovered. OX14 is a novel bisphosphonate that is very potent and has lower bone-binding properties than current bisphosphonates [110]. Lidadronate via the mevalonate pathway and OX14 both show promise for osteoporosis treatment; however, limited studies mean more evidence is needed [107,109,110].

Denosumab is a monoclonal antibody that is anti-RANKL neutralising and has recently been developed for use in osteoporosis treatment [12]. RANKL’s role is to regulate bone resorption using osteoclasts, and denosumab inhibits this, making it a potent treatment for osteoporosis [12]. Denosumab effectively binds to RANKL without interacting with tumour necrosis factor ligands. Patients taking denosumab have shown an increase in BMD and reduced bone resorption [12]. Clinical trials, therefore, strengthen the evidence available for using denosumab in osteoporosis. Long-term studies have demonstrated denosumab to be safe and effective for up to 10 years of use [12].

When compared with bisphosphonates, denosumab showed a greater increase in BMD [12]. This improvement was recorded in patients newly diagnosed with osteoporosis and in patients who switched from bisphosphonates to denosumab [12]. This is because denosumab preserves modelling-based bone formation (MBBF) but inhibits remodelling-based bone formation (RBBF), thus leading to enhanced BMD [12]. As serum denosumab concentrations decrease, MBBF is still preserved, and RBBF slightly increases. This helps conserve bone strength because of a further increase in BMD, and any damaged bone is replaced [12].

Osteocytes increase RANKL expression in bone resorption [12]. Bone resorption is stimulated in areas of increased RANKL, as RANKL binds to its RANK receptor [12]. Denosumab inhibits bone resorption by binding to RANKL and preventing it from stimulating bone resorption by binding to RANK. Another benefit of denosumab is that it only needs administration twice a year due to its long half-life. Denosumab is, therefore, effective in increasing BMD and reducing fracture risk, and its benefits may be enhanced by administering it following treatment with bisphosphonates [12].

The second group of drugs used in osteoporosis are anabolic drugs, and, unlike anti-resorptive drugs, they work by promoting osteogenesis instead of preventing bone loss [13]. They are highly recommended in PMOP, such as WNT antagonists [102]. Anti-sclerostin monoclonal antibodies and anti-DKK1 monoclonal antibodies are inhibitors of WNT antagonists and are used to treat osteoporosis by promoting osteogenesis [111]. Sclerostin was discovered because inactivating mutations of *SOST* lead to increased bone mass [14]. This means that inhibiting sclerostin could be used to enhance BMD [14].

In clinical trials, rats with PMOP were treated with anti-sclerostin monoclonal antibodies and prevention of bone loss and increased BMD was recorded [112]. Romosozumab (AMG-785) is the first anti-sclerostin monoclonal antibody that has been shown to enhance osteogenesis in humans and is especially effective in PMOP [14,113].

The use of anti-DKK1 monoclonal antibodies for treatments is undergoing trials; however, it has been shown to prevent bone loss and increase osteogenesis [98]. Furthermore, an increase in WNT signalling using these therapies has been linked with malignancies, including cancer [114]. This means that patients on these therapies require regular monitoring [115].

Calcilytic agents are also used in anabolic therapy as they help with osteogenesis [13]. They work by mimicking hypocalcaemia and induce the secretion of parathyroid hormone. Currently, careful monitoring is essential because sustained parathyroid hormone secretion can worsen bone disease [13]. Scientists are, therefore, working on better calcilytic agents [116,117]. MK-5442, a recently investigated calcilytic agent, has shown promise in stimulating pulsatile parathyroid hormone release and osteogenesis, although it is currently not yet widely approved [118].

## Conclusions

This literature review explores the complex genetic landscape underlying osteoporosis, encompassing both monogenic and multifactorial causes. Mutations in genes such as *LRP5*, *COL1A1*, and *GORAB* contribute to impaired bone formation and reduced BMD, with syndromic forms often presenting more severe clinical manifestations. While most monogenic osteoporosis is autosomal recessive, emerging evidence suggests that heterozygous mutations may also lead to milder phenotypes, underscoring the need for further investigation into genotype-phenotype correlations. Multifactorial osteoporosis, particularly in postmenopausal women, involves numerous low-frequency loci that collectively influence bone fragility. Identifying and classifying these genetic contributors are essential for advancing early detection and personalised care. Tailoring treatment to a patient’s genetic profile may significantly improve therapeutic outcomes. Current therapies, including anti-resorptive and anabolic agents, remain the mainstay, though concerns exist regarding long-term safety, especially in treatments targeting WNT signalling. Alternatives, such as modulation of the mevalonate pathway, offer promising potential with fewer systemic effects. Future directions include comparing existing pharmacological agents with emerging therapies and exploring genetic modification techniques as a curative approach. As osteoporosis prevalence continues to rise with ageing populations, investment in predictive screening tools and targeted interventions will be key to reducing disease burden and preserving quality of life.
